# An Analysis of Ocular Biometrics: A Comprehensive Retrospective Study in a Large Cohort of Pediatric Cataract Patients

**DOI:** 10.3390/jcm13164810

**Published:** 2024-08-15

**Authors:** Luca Schwarzenbacher, Lorenz Wassermann, Sandra Rezar-Dreindl, Gregor S. Reiter, Ursula Schmidt-Erfurth, Eva Stifter

**Affiliations:** 1 Department of Ophthalmology and Optometry, Medical University of Vienna, Währinger Gürtel 18-20, 1090 Vienna, Austria; luca.schwarzenbacher@meduniwien.ac.at (L.S.);; 2 Laboratory for Ophthalmic Image Analysis, Medical University of Vienna, Spitalgasse 23, 1090 Vienna, Austria

**Keywords:** pediatric cataract, pediatric cataract surgery, ocular biometry, IOL placement, surgical planning, corneal diameter

## Abstract

**Objectives:** This study aims to provide a comprehensive analysis of ocular biometric parameters in pediatric patients with cataracts to optimize surgical outcomes. By evaluating various biometric data, we seek to enhance the decision-making process for intraocular lens (IOL) placement, particularly with advanced technologies like femtosecond lasers. **Methods:** This retrospective comparative study included pediatric patients with cataracts who underwent ocular biometric measurements and cataract extraction with anterior vitrectomy at the Medical University of Vienna between January 2019 and December 2021. Parameters measured included corneal diameter (CD), axial length (AL), corneal thickness (CT) and flat and steep keratometry (Kf and Ks). The study explored the correlations between these parameters and IOL placement. **Results:** A total of 136 eyes from 68 pediatric patients were included in the study. Significant positive correlations were found between corneal diameter, age and AL. The mean CD was 11.4 mm, mean AL was 19.5 mm, CT was 581.2 ± 51.8 µm, Kf was 7.76 ± 0.55 mm and Ks 7.41 ± 0.59 mm, respectively. Older pediatric patients with larger corneal diameters and longer ALs were more likely to receive in-the-bag IOL implantation. Conversely, younger patients often required alternative IOL placements or remained aphakic. Our data indicated that over 95% of the study population and all patients aged one year and older had a corneal diameter of 10 mm or larger. **Conclusions:** Detailed ocular biometric analysis is crucial for optimizing both surgical outcomes and postoperative care in pediatric cataract patients. The positive correlations between CD, age and AL underline the importance of individualized surgical planning tailored to each patient’s unique anatomical features. Additionally, our findings suggest that the use of a femtosecond laser is both feasible and safe for pediatric patients aged one year and older, potentially offering enhanced surgical precision and improved outcomes.

## 1. Introduction

Congenital and acquired lens opacities are one of the most important causes of preventable blindness in children worldwide, requiring early surgical intervention to prevent long-term visual impairment [1].

The success of these interventions is highly dependent on reliable and reproducible ocular biometric measurements [2]. Accurate biometry forms the foundation for several critical aspects of managing congenital cataracts. These measurements not only serve as a basis for follow-up examinations and intraocular lens (IOL) calculations but are also crucial for assessing potential glaucoma risks, especially in cases where there is a significant increase in axial length [3]. Furthermore, they are vital for predicting refractive development in general, including the calculation of spectacles and contact lenses for non-cooperative patients. Additionally, biometry is crucial for all subsequent clinical decisions, such as planning the next examination under anesthesia or determining the optimal timing for secondary IOL implantation.

The measurement of ocular biometrics including axial length (AL), corneal curvature, anterior chamber depth (ACD) and lens thickness (LT) is essential for the accurate calculation of IOL power and surgical planning. The additional use of advanced imaging technologies, such as swept-source optical coherence tomography (SS-OCT) incorporated in femtosecond lasers, has significantly improved the precision of diagnostics, thereby facilitating superior surgical outcomes [4]. This is of particular interest in exceptionally short or long eyes and for evaluating ocular development in pediatric patients [5].

In the pediatric population, age and anatomical factors significantly influence biometric variations, which in turn affect the choice and positioning of IOLs. While it is well established that axial elongation, corneal curvature and age profoundly impact refractive outcomes, there is limited knowledge regarding the specific effects of corneal diameter and axial length on the success of in-the-bag IOL placements [6].

Larger corneal diameters and longer axial lengths are generally associated with more favorable in-the-bag IOL placements, providing better structural support and stability. However, in cases of congenital cataract patients with ocular malformations, this is often not the case. Such anomalies can lead to smaller corneal diameters and shorter axial lengths, potentially complicating the placement and stability of in-the-bag IOLs. Therefore, it is crucial to perform accurate eye examinations and biometry to ensure proper assessment and planning for these patients [7]. Accurate biometry is essential for making informed decisions on the most suitable surgical approach and IOL placement, particularly when dealing with anatomical irregularities [8].

The aim of this study is to provide an in-depth analysis of ocular biometric parameters in pediatric patients with cataracts, thus improving our understanding of the factors that influence surgical outcomes and the decision-making process for IOL placement. Furthermore, by evaluating a wide range of biometric data during challenging examinations under anesthesia, we aim to optimize preoperative planning, intraoperative techniques and postoperative care for pediatric cataract patients. This research is crucial for informing the parents of patients about the expected outcomes and surgical options, ensuring that the best possible care is provided. Moreover, the study aims to validate the safety of femtosecond laser-assisted cataract surgery in this population.

## 2. Materials and Methods

In this retrospective analysis, we evaluated the medical records of patients who underwent pediatric cataract extraction for uni- or bilateral cataracts at the Medical University of Vienna between January 2019 and December 2021. Approximately half of these patients were left aphakic, while the other half received primary IOL implantations. The IOLs were placed either in the bag, in the sulcus or as iris-fixated lenses. The decision regarding IOL implantation was determined based on the anatomical constraints and specific needs of each patient. Additionally, data from unaffected control eyes were included in our study.

The study received approval from the Ethics Committee of the Medical University of Vienna (approval number 1204/2020) and was conducted in accordance with the principles of the Declaration of Helsinki. Due to the retrospective design of the research, the requirement for informed consent was waived.

### 2.1. Examinations

Patients underwent a standard ophthalmologic examination under anesthesia directly before cataract surgery including the unaffected partner-eye if congenital cataract was unilateral. Our examinations included applanation tonometry (indentation tonometry type Schiötz), white-to-white measurement (surgical caliper), corneal thickness (pachymeter, Quantel Medical Pocket II, Lumibird Medical, Cournon-d’Auvergne, France), and axial length (ultrasound a-scan, Quantel Medical Aviso, Lumibird Medical, Cournon-d’Auvergne, France); flat keratometry (Kf) and steep keratometry (Ks) were measured with a hand-held refractometer (Retinomax 5, Bon Optic, Lübeck, Germany). The examinations were conducted in a standardized manner, commencing with keratometry measurements and subsequently proceeding to the remaining parameters that necessitate direct eye contact, such as the ultrasound A-scan, which was always conducted last. This sequence was implemented to ensure that each measurement did not negatively impact the subsequent ones, maintaining the accuracy and reliability of the data collected.

### 2.2. Statistical Analysis

The objective of the statistical evaluation of our dataset was to elucidate the relationships between corneal diameter, age, and axial length. Additionally, the relationship between age, axial length and the IOL implantation (in-bag vs. in-sulcus vs. aphakia vs. iris-fixated) was investigated. The preliminary analyses included descriptive statistics aimed at summarizing the central tendency, dispersion, and shape of the distribution for each variable. A mixed-effects model was calculated to investigate the impact of age, axial length, sex and the interaction between age*axial length and corneal diameter. Another mixed-effects model was calculated to investigate the impact of age, axial length, sex and the interaction between age*axial length and corneal thickness. In light of the paired nature of the data, with two eyes per patient, inter-eye correlation was accounted for as a random factor in the mixed-effects models, thus avoiding any violation of the independence assumption inherent in most statistical tests. To investigate the impact of astigmatism, a mixed-effects model was constructed with corneal diameter as the fixed effect. To investigate the impact of age and axial length on the implantation of the lens, a multinominal logistic mixed-effects model was calculated with “no surgery” set as the reference category. To investigate the impact of age and axial length on the type of lens implantation (bag, sulcus, iris, aphakia), a multinominal logistic mixed-effects model was calculated with “no surgery” as the reference category.

All statistical tests were two-tailed, and a *p*-value of less than 0.05 was considered to indicate statistical significance. The analyses were performed using a statistical software package (SPSS 28.0). The rigor of our statistical approach ensured the validity and reliability of the conclusions drawn from our study on the interrelationships among corneal diameter, age and axial length in pediatric patients with cataract.

## 3. Results

Between January 2019 and December 2021, this study included 136 eyes from 68 patients, comprising 34 males and 34 females.

Table 1 displays the baseline characteristics, summarizing data on age, axial length, corneal diameter, corneal thickness, and both flat and steep keratometry readings.

**Figure 1:**
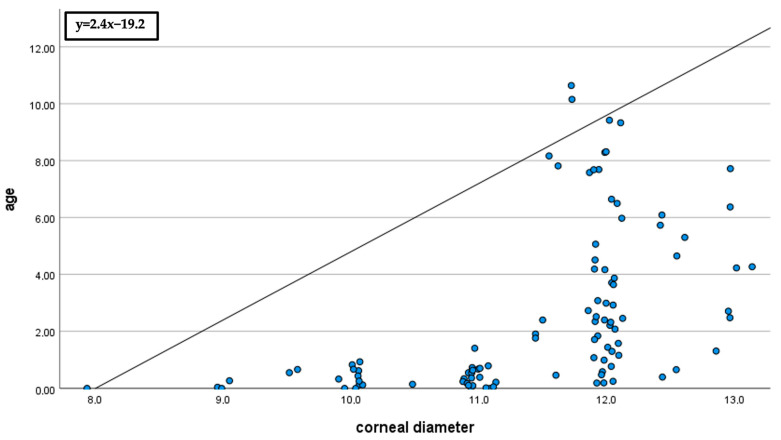
Correlation of corneal diameter to age (r = 0.7). Bivariate scatter plot of age (years) and corneal diameter (mm). The scatter plot demonstrates the positive correlation of corneal diameter with age.

**Figure 2:**
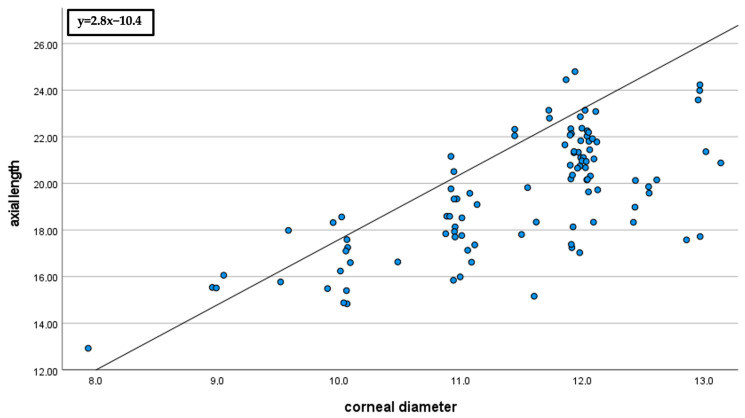
Correlation of corneal diameter to axial length (r = 0.6). Bivariate scatter plot of axial length (mm) and corneal diameter (mm). The scatter plot demonstrates the positive correlation of corneal diameter with axial length.

The correlation of corneal diameter to age and axial length was significantly positive (*p* < 0.01). The Spearman–Rho correlation coefficient (r) was 0.7 for age and 0.6 for axial length (see Figure 1 and Figure 2).

In Table 2, we evaluated the number of patients who received intraocular lenses (IOLs) versus those left aphakic, considering age and axial length. We also incorporated data from healthy control eyes.

The multinominal logistic mixed-effects model showed a significant positive coefficient of 0.314 for age (*p* < 0.01) in cases of in-the-bag implantation, suggesting that in-the-bag IOL placement is associated with higher age compared to the control. In contrast, for sulcus implantation, we observed a negative coefficient of −1.98 (*p*-value of 0.075), indicating a trend where decreased age increases the probability of sulcus placement for the IOL.

**Figure 3:**
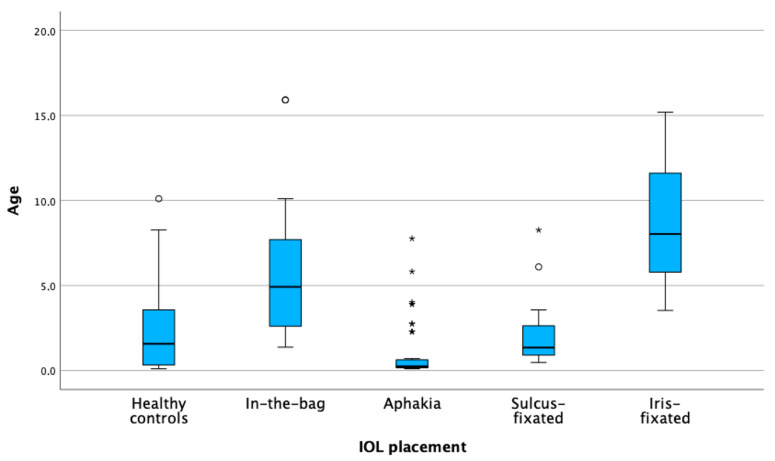
IOL implantation dependent on age. Box plots of the distribution of age across different intraocular lens (IOL) placement groups. The asterisks (*) mark extreme outliers more than 3 times the interquartile range and circles (o) indicate mild outliers.

The analysis for the iris-fixated lens revealed an age coefficient of −0.131 with a *p*-value of 0.700 in a rather low number of cases, showing that age does not significantly impact the decision for iris-fixated IOL placement. For scenarios involving no IOL implantation, the age coefficient was −0.360 with a *p*-value of 0.032, suggesting that a younger age significantly raises the chances of opting out of IOL implantation following cataract extraction. Figure 3 is a visual representation of the data, categorized by age.

**Figure 4:**
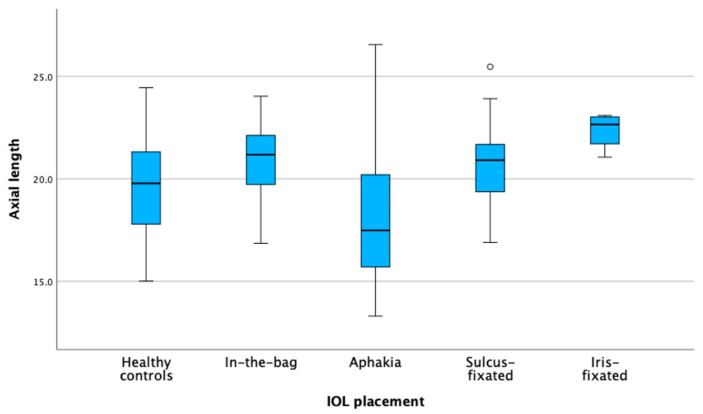
IOL implantation dependent on axial length. Box plots of the distribution of axial length across different intraocular lens (IOL) placement groups. The circle (o) indicates a mild outlier between 1.5 and 3 times the interquartile range.

**Figure 5:**
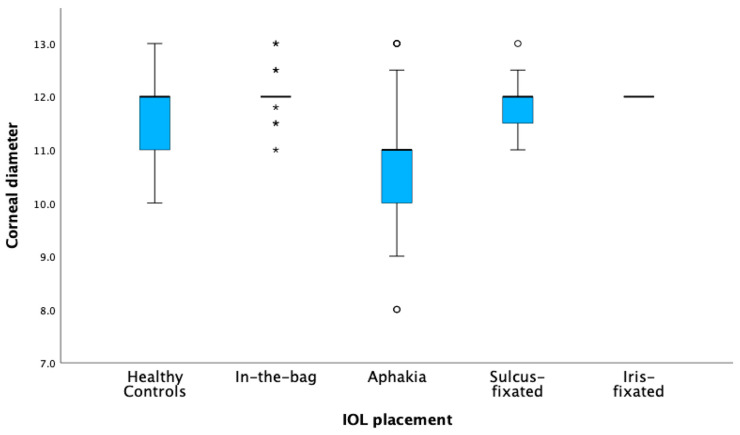
IOL implantation dependent on corneal diameter. Box plots of the distribution of corneal diameter across different intraocular lens (IOL) placement groups. The asterisks (*) mark extreme outliers more than 3 times the interquartile range and circles (o) indicate mild outliers.

The multinomial logistic regression model dependent on axial length shows that none of the coefficients are statistically significant as all *p*-values are well above the conventional threshold of 0.05. Figure 4 is a visual representation of the data, categorized by axial length, with Figure 5 categorizing by corneal diameter.

Table 3 shows the specific age and axial length characteristics dependent on the IOL localization after cataract extraction, as well as the healthy control characteristics.

## 4. Discussion

In the present study, we conducted an in-depth analysis of ocular biometric parameters in patients with pediatric cataracts aged from 5.5 weeks to 15.9 years and explored factors influencing surgical outcomes and the decision-making process for IOL placement.

Our findings revealed several significant correlations and trends, providing relevantly increased insights into optimizing surgical strategies for the treatment of pediatric patients with cataract.

Our data indicate a positive correlation between corneal diameter, age and axial length. The mean corneal diameter was 11.4 mm, while mean axial length was measured at approximately 19.5 mm. The correlation between axial length growth and age is well established in the literature [9,10]. Given the strength of this correlation, age serves as a reliable proxy for axial length in many clinical scenarios, thereby further supporting its use as a primary variable in our analysis. The data presented in our study demonstrate a high degree of comparability to adult biometric data, with axial length values averaging approximately 23.8 mm in adults aged 40 to 80 years based on comparable data from a large population-based health study [11].

In contrast, this degree of comparability is not fully reflected in the optical diameter, which has an average of 11.4 mm in our pediatric cohort and 12.2 mm in the adult cohort. In other words, this represents an increase of approximately 7%, while the increase in axial length is approximately 22% from a pediatric population compared to an adult population [11]. Nevertheless, when the entire surface area from limbus to limbus is considered, this increase is of great influence. The area in the pediatric cohort is 102.1 mm^2^, compared to 116.9 mm^2^ in adults, representing a 14.5% increase. Additionally, the curvature ratios change significantly, affecting the refractive power of the cornea [9]. These changes are interconnected and contribute to the natural process of emmetropization. Surgical intervention can have a substantial impact on this process [12].

Regarding IOL implantation, we found a significant positive correlation between higher age and successful in-the-bag IOL placements. This finding aligns with the notion that older patients, who generally have larger corneal diameters and longer axial lengths, are more likely to benefit from in-the-bag IOL placements due to better structural support and stability [13]. Conversely, younger patients, who tend to have smaller corneal diameters and shorter axial lengths, are more likely to require alternative IOL placements, such as sulcus-fixated IOLs, or remain aphakic. This is evident in our findings, which align with the understanding that the capsular bag often lacks sufficient stability in younger and shorter eyes. Additionally, the refractive shift observed is a natural consequence of the eye’s growth and emmetropization process [14,15]. Preoperative biometry helps to accurately assess the anatomical conditions of the eye, which is crucial for surgical planning. It is important for the surgeon to have a clear understanding of these conditions before commencing the procedure.

The use of iris-fixated IOLs in pediatric cataract patients showed a tendency towards older patients, all of whom were diagnosed with Marfan syndrome, having longer axial lengths. However, the data presented are not representative of this cohort, as fewer iris-fixated IOLs were provided compared to the other cohorts. The trend seen in our data is consistent with the broader understanding in the literature of the importance of tailored surgical approaches based on individual biometric profiles [5].

There is a lack of consensus in the literature regarding the necessity of primary IOL implantation in patients with congenital cataracts under the age of two years. This is particularly evident in the context of long-term visual acuity, with particular focus on visual axis opacification (VAO) and posterior synechiae [16,17]. Nevertheless, there are advantages to primary IOL implantation, including a reduced risk of aniseikonia in unilateral cases and constant optical correction, even if less care is given. We believe that, regardless of the approach to IOL implantation, anterior vitrectomy is essential for lensectomy in congenital cataract patients. A study by Kaur et al. supports this approach, demonstrating a reduction in VAO and complications when posterior optic capture was performed compared to in-the-bag implantation [18]. All of our cases underwent anterior vitrectomy, and no complications were observed, except for a case with congenital PFV syndrome and an intraoperative retinal detachment in an 8-week-old unilateral cataract patient that required gas tamponade in the same procedure.

In our dataset, we identified an extreme outlier of axial length in the aphakia group. This was a three-year-old with an acquired cataract that was not associated with trauma, metabolic disorders or other comorbidities such as glaucoma or high myopia. Nevertheless, our multinomial logistic regression model revealed that age emerged as a significant predictive factor, whereas axial length did not show statistical significance. This discrepancy may be attributed to the considerable variability in axial length and the statistical phenomenon of collinearity. Collinearity arises when two or more predictor variables in a regression model are highly correlated, as is the case with age and axial length. This can result in unstable estimates of the coefficients, making it challenging to ascertain the individual impact of each predictor on the outcome variable.

The findings of our study indicate that corneal diameter in patients with pediatric cataracts is generally sufficient for the docking procedure for femtosecond laser-assisted cataract surgery (FLACS). To ensure the safety of the docking process, we recommend a minimum corneal diameter of 10 mm, which can be seen in over 95% of our study population and in all patients aged one and older. The data suggest that the majority of pediatric cataract patients are suitable for FLACS, regardless of the chosen IOL approach. The use of femtosecond laser technology is becoming increasingly relevant in pediatric cataract surgery, particularly for the precise creation of anterior and posterior capsulotomy, especially when planning to implant a bag-in-the-lens IOL [19,20].

Our study has various limitations that should be acknowledged. First, the retrospective design and potential selection biases in patient inclusion could impact the generalizability of our findings. Additionally, the uneven distribution of patient groups, particularly the small sample size in the iris-fixated IOL group, limits the strength of conclusions that can be drawn for this subgroup. Furthermore, the lack of data on long-term visual acuity and refractive outcomes restricts our ability to fully assess the clinical impact of the biometric findings and the surgical decisions made based on them. Another consideration is the time gap between data collection (January 2019 to December 2021) and the publication of this study in 2024. The extended analysis period was due to delays associated with the COVID-19 pandemic, which impacted research timelines. Despite this gap, there have been no significant changes in clinical or surgical practices in our department. Therefore, we believe that the biometric data remain comparable with current measurements and are still relevant for today’s clinical practice. Future studies should aim to address these limitations by including a larger patient population and by providing long-term follow-up data to better evaluate clinical outcomes.

In conclusion, our study substantiates the importance of detailed ocular biometric analysis in pediatric patients with cataracts to optimize surgical outcomes. The positive correlations between corneal diameter with age and axial length enhance our understanding of how these factors influence the decision-making process for IOL placement, as well as intraoperative surgical approaches and postoperative care. Our findings suggest that older pediatric patients with larger corneal diameters and longer axial lengths are more likely to benefit from in-the-bag IOL implantation, which provides better structural support and stability. These insights should be considered when informing parents to ensure the provision of optimal care for their children. Conversely, younger patients may require alternative IOL placements or remain aphakic due to their smaller ocular dimensions. The data also highlight the feasibility and safety of utilizing FLACS in this population, with the majority of patients having sufficient corneal diameter for the procedure. Furthermore, our study reinforces the necessity of anterior vitrectomy in reducing complications such as visual axis opacification, aligning with existing evidence of its benefits. Future research should continue to refine pre- and intraoperative strategies, taking into account long-term outcomes and the evolving technological advancements in cataract surgery.

## Figures and Tables

**Table 1:** Patient characteristics.

Variables	Mean ± SD	Range
Age (y)	3.5 ± 4.3	0.1 to 15
Sex (m/f)	34/34	-
Axial Length (mm)	19.55 ± 2.77	13.3 to 26.55
Corneal Diameter (mm)	11.43 ± 1.06	8 to 13
Corneal Thickness (µm)	581.15 ± 51.78	416 to 728
Kf (mm)	7.76 ± 0.55	6.18 to 9.07
Ks (mm)	7.41 ± 0.59	5.70 to 8.61

Kf = flat keratometry; Ks = steep keratometry. The data are presented as mean ± standard deviation (SD).

**Table 2:** IOL status after cataract surgery.

	IOL Implantation	Aphakia	Healthy Control
n	52	48	36
Age (y)	5.00 ± 4.02 [0.5; 16]	1.00 ± 1.63 [0.1; 7.7]	2.55 ± 2.81 [0.1; 10]
Axial Length (mm)	20.93 ± 1.99 [16.8; 25.5]	18.28 ± 3.11 [13.3; 26.6]	19.57 ± 2.24 [15.0; 24.45]

IOL = Intraocular lens. The data are presented as mean ± standard deviation (SD) and the range (minimum to maximum).

**Table 3:** IOL placement.

	Healthy Controls	In-The-Bag	Aphakia	In Sulcus	Iris-Fixated
n	36	36	48	10	6
Age (y)	2.55 ± 2.81 [0.1; 10.1]	5.75 ± 3.90 [1.38; 15.91]	1.00 ± 1.63 [0.1; 7.7]	2.32 ± 2.36 [0.5; 8.3]	8.69 ± 4.82 [3.5; 15.2]
Axial Length (mm)	19.57 ± 2.24 [15.0; 24.5]	20.78 ± 1.91 [16.9; 24.0]	18.28 ± 3.11 [13.3; 26.6]	20.82 ± 2.30 [16.9; 25.5]	22.37 ± 0.93 [21.1; 23.1]

The data are presented as mean ± standard deviation (SD) and the range (minimum to maximum).

## Data Availability

Data are available upon reasonable request.
